# Status Quo of Progress Testing in Veterinary Medical Education and Lessons Learned

**DOI:** 10.3389/fvets.2020.00559

**Published:** 2020-08-21

**Authors:** Lisa Herrmann, Christina Beitz-Radzio, Dora Bernigau, Stephan Birk, Jan P. Ehlers, Birte Pfeiffer-Morhenn, Ingrid Preusche, Andrea Tipold, Elisabeth Schaper

**Affiliations:** ^1^Centre for E-Learning, Didactics and Educational Research (ZELDA), University of Veterinary Medicine Hannover, Foundation, Hanover, Germany; ^2^Faculty of Veterinary Medicine, Ludwig-Maximilians-Universität Munich, Munich, Germany; ^3^Faculty of Veterinary Medicine, Leipzig University, Leipzig, Germany; ^4^Faculty of Veterinary Medicine, Freie Universität Berlin, Berlin, Germany; ^5^Didactics and Educational Research in Health Science, Faculty of Health, Witten/Herdecke University, Witten, Germany; ^6^Veterinary Medical Faculty of Justus Liebig University Giessen, Giessen, Germany; ^7^Centre for Study Affairs, University of Veterinary Medicine, Vienna (Vetmeduni Vienna), Vienna, Austria; ^8^Small Animal Clinic, Neurology, University of Veterinary Medicine Hannover, Foundation, Hanover, Germany

**Keywords:** E-assessment, veterinary education, day 1 competencies, feedback instruments, curriculum evaluation, longitudinal testing, electronic online test, multiple choice question (MCQ)

## Abstract

Progress testing is an assessment tool for longitudinal measurement of increase in knowledge of a specific group, e.g., students, which is well-known in medical education. This article gives an overview of progress testing in veterinary education with a focus on the progress test of the German-speaking countries. The “progress test veterinary medicine” (PTT) was developed in 2013 as part of a project by the Competence Centre for E-Learning, Didactics and Educational Research in Veterinary Medicine—a project cooperation of all German-speaking institutes for veterinary medicine in Germany, Austria, and Switzerland. After the end of the project, the PTT was still continued at six locations, at each of the five German schools for veterinary medicine and additionally in Austria. Further changes to the PTT platform and the analysis were carried out to optimize the PTT for continuing to offer the test from 2017 to 2019. The PTT is an interdisciplinary, formative electronic online test. It is taken annually and is composed of 136 multiple-choice single best answer questions. In addition, a “don't know” option is given. The content of the PTT refers to the day 1 competencies described by the European Association of Establishments for Veterinary Education. The platform Q-Exam® Institutions (IQuL GmbH, Bergisch Gladbach, Germany) is used for creating and administrating the PTT questions, the review processes and organizing of the online question database. After compiling the test by means of a blueprint, the PTT file is made available at every location. After the last PTT in 2018, the link to an evaluation was sent to the students from four out of these six partner Universities. The 450 analyzed questionnaires showed that the students mainly use the PTT to compare their individual results with those of fellow students in the respective semester. To conclude our study, a checklist with our main findings for implementing progress testing was created.

## Introduction

Progress tests (PT) are well-known in medical education (1–5). Lately, PTs have been applied in related fields like dentistry (6), psychology (7), or veterinary medicine (8, 9).

In veterinary medicine, PTs are conducted in Europe in Utrecht, the Netherlands (9) and in German-speaking countries where the test is called “progress test veterinary medicine” (PTT, Progress Test Tiermedizin) (8).

Progress testing is a tool for longitudinal measurement of increase in knowledge of a group, e.g., students from a whole University, a single semester or a specific class (10). During longitudinal testing, an observed group takes several comparable tests at regular intervals (11). Distinguishing features of PTs are test blueprints and periodical testing (12). A test blueprint is a concept to assign question items to subjects and to create a weighting of these subjects. Progress testing can be carried out in a summative form (grading of the test results) as well as a formative test (without grading) (13) and has a varying item repertoire for every test (12). The items are multiple choice (MC) questions (14). That means there is only one right answer (attractor) but various wrong answers (distractors).

In addition, a “don't know” option should prevent guessing and reduces accidental mistakes (15). This “don't know” option helps those students unable to answer the questions because they have not yet attended the respective classes or cannot remember what they have learnt. Indeed, students should show confidence in their chosen answer. The “don't know” option will start a process of reflection: What do I know? Vs. What do I not know (8)?

Different purposes of progress testing are described in the literature: It can function as a feedback instrument for students, show spontaneously accessible knowledge, measure the increase in knowledge or serve as a tool for comparing different curricula (14). The main objective for developing and implementing PTs is to promote sustainable knowledge and to stop assessment-steered learning (1, 14). As students mostly just focus on acquiring a large amount of knowledge (8) and proceed with rote learning, it is interesting to examine sustainable knowledge (7). It is discussed that common summative exams encourage the so-called binge learning (10). The impact of progress testing on students' learning routine would be an interesting topic to examine. Another main objective was to implement a quality management tool for curricula (1).

Progress testing in medical studies was independently created at two separate Universities. In the 1970s, the University of Limburg, Maastricht, the Netherlands (14) and the School of Medicine of the University of Missouri, Kansas City, USA (1) developed the first PTs. Different reasons for establishing such tests were mentioned.

The School of Medicine of the University of Missouri needed an evaluation tool for the curriculum and new indicators to test the development of students' knowledge (1). In comparison, the University of Limburg wanted to prevent students learning only for specific tests without any continuance of knowledge (14).

Following the lead set by the University of Limburg, Maastricht, students of the Charité-Berlin University of Medicine, Berlin, Germany (Charité-Universitätsmedizin Berlin) were responsible for introducing progress testing in Germany in 2000 (3). The main purposes for this use of progress testing were a need for more individual feedback on performance and level of education as well as the comparison of the old and new curricula at the Charité (3). Meanwhile, a cooperation between German and Austrian Universities was established in order to jointly organize and carry out the German medical PT (Progress Test Medizin, PTM) (16).

The expansion of medical progress testing throughout Europe led to several test designs, which we will classify exemplarily in the following.

### Single or Composite Project

While PTs in the German-speaking countries are carried out across several Universities (4), several pilot projects, as in Tampere, Finland are conducted only at a single University to improve curricular adjustments (17).

### Frequency

The average test frequency is twice a year as is the case with the PTM (3), the University of Limerick, Limerick, Ireland (12) or the psychological PT of Witten/Herdecke University, Witten, Germany (7). Furthermore, some Universities perform PTs once a year, e.g., the competency-based formative PT with student-generated MC questions, Ruprecht Karls University of Heidelberg, Heidelberg, Germany (18) or up to four times a year as in the Dutch medical PT (19) and for the Bachelor of Medicine and Bachelor of Surgery Courses at the University of Plymouth, Plymouth, England (20).

### Content

The basis for the question pool covers all the required medical knowledge to quality as a doctor (“day 1 competences”) (2, 3, 12, 17). To test only particular subjects, the question pool can be reduced according to the syllabus. Those reduced PTs are used at the Ludwig Maximilian University of Munich, Munich, Germany for the subject Internal Medicine (5) or at the University of Ulm, Ulm, Germany for the Dentistry PT in Anatomy (21).

### Participation

Mostly the participation is compulsory for all students (14, 17, 22) but there are combinations of compulsory and voluntary tests. The participation in the PTM for example is optional for the preclinical years and obligatory for those students in the clinical phase (3, 4). The reason is that students in earlier semesters are most likely to be disappointed by their lack of increase in knowledge (4). Especially formative PTs, e.g., the competency-based formative PT with student-generated MC questions or the dentistry PT in Anatomy, require no compulsory participation (18, 21).

### Rating

The University of Limburg, Maastricht, the Netherlands and McMaster University, Hamilton, Ontario, Canada carry out summative PTs (12, 19). Nevertheless, the formative aspect of progress testing is maintained because the students receive additional feedback on their performance (12, 23). In comparison, the PTM and the psychological PT are used for individual formative feedback only (3, 7).

### Question Types

Most of the universities performing PTs use single-best answer MC questions with one right answer and an average of two-three distractors (13, 17, 21). Some more recent progress tests, like the Psychological PT and the Dentistry PT in Anatomy tests with the true/false format use three to five answers (7, 21). Regardless of the question type, a “don't know” option is added in most cases (12), whereas the University of Manchester, Manchester, England or the Dutch Radiology PT are missing this option (12, 22).

### Availability

At the outset, PTs were carried out as paper-based tests (4, 12, 14). However, more recent literature describes the implementation of electronic tests, e.g., in the PTM at the University of Cologne, Cologne, Germany (4), the Dutch Radiology PT (22) and the competency-based formative PT with student-generated MC questions at the University of Heidelberg (24).

### Number of Questions and Open Time

The number of questions varies from 100 (7, 21) to 130 (12, 24) and right up to 200 questions (19, 22, 25). The time available to complete the test differs from 50 min (21) to two and a half hours (12) and up to 4 h (12, 17, 25). This results in an average processing time of 1 min per question. As part of the formative aspect, students have had no time limit to complete the competency-based formative PT with student-generated MC questions at the University of Heidelberg since 2018 (26).

### Test Results

The availability of the test results differs considerably between the universities. The Berlin University of Medicine sends individual results to the participants only (3). Besides, the authors of the questions receive statistical data, e.g., responses, statistical power and item difficulty (3). Feedback for students for the psychological PT and the PT with student-generated MC questions is comparable to PTM (7, 18). However, in addition the departments or Universities are informed about the performance of the prevailing cohorts broken down into subjects (7, 18). The individual results from the Dutch Radiology PT are send to the participants but also to their supervisors (22). The results of their own residents are also compared with the other participating training centers in the Netherlands (22).

This study presents an overview of the use of progress testing in veterinary medical education and established the spread of PTs in veterinary medicine over Europe. Furthermore, the opinion of the students of the German speaking Universities on a PTT was determined.

## Materials and Methods

### Study on Progress Testing in European Veterinary Medical Education

Progress testing in veterinary medical education has gained increasing importance in Europe over the last 10 years (8, 9). To gain more knowledge concerning the distribution of progress testing at European veterinary schools, a literature search and an online survey were carried out. In addition to identifying further PTs, the structure and performance types of other PTs were investigated.

Literature search was performed using online databases ResearchGate GmbH, National Center for Biotechnology Information (NCBI), PubMed Central® and ScienceDirect®, Google Scholar, LIVIVO—ZB Med Search Portal for Live Sciences and the search engine VetSearch from the University of Veterinary Medicine Hannover, Foundation, Hanover, Germany (TiHo).

Keywords included the terms *progress test, progress testing, progress test in veterinary medicine* and *veterinary medical/medical education*—also translated into German. In addition, the bibliographies of available publications were scanned.

The questionnaire was created with the online software LimeSurvey® (LimeSurvey GmbH, Hamburg, Germany) and sent to 82 European Universities. The descriptive analysis was carried out with Microsoft® Office Excel 2010 (Microsoft Corporation, California, USA). Data were collected in compliance with the privacy policy Art. 6 I lit. e in conjunction with 89 GDPR, § 3 I 1 No. 1 NHG, § 13 NDSG (Lower Saxony Data Protection Act).

### PTT Evaluation

Progress testing in German-speaking countries was implemented in 2013 as part of a project cooperation of all German-speaking institutes for veterinary medicine in Germany, Austria and Switzerland. After the end of the project, the PTT was still continued annually in Germany and in Austria.

After the last PTT in December 2018, a link to an online questionnaire was sent to the students from four of the six partner Universities. The questionnaire was created and operated with the online software LimeSurvey® (LimeSurvey GmbH, Hamburg, Germany). The students at all four participating Universities received an invitation email with the link to the survey. Data were collected in compliance with the privacy policy Art. 6 I lit. e in conjunction with 89 GDPR, § 3 I 1 No. 1 NHG, § 13 NDSG (Lower Saxony Data Protection Act).

The questions related to the participation in the last PTT and the reasons for or against participating in the test. Furthermore, the students were able to rate statements about the individual benefit of the PTT. The participation was voluntary and anonymous.

### Statistical Analysis

The descriptive analysis was carried out using Microsoft® Office Excel 2010 (Microsoft Corporation, California, USA).

The non-parametric analysis was carried out with SAS® Enterprise Guide® 7.1 (SAS Institute Inc., Cary, USA). The Chi^2^-test was chosen as suitable for testing the hypothesis that there was no association between the year of study and the form of responses to questions and statements as shown in Figures 7, 8 and Table 3A. The sample size included 450 participants from four German-speaking schools for veterinary medicine. The significance level was 5%. If *p*-value was <0.05, a significant association between the year of study and form of responses was indicated.

In order to obtain valid test results, the following response options were combined: “Totally agree” and “Rather agree” were summarized to “Agree” and “Do not agree at all” and “Do rather not agree” were combined to the term “Do not agree” (Figure 7).

### Ethics Statement

This study was conducted according to the ethical standards of the University of Veterinary Medicine Hannover, Foundation. The doctoral thesis committee of the University, which acts as the University's ethics committee, validated the project in accordance to ethical guidelines regarding research with human participants and approved the study. Written consent to be part of the study was obtained from all participants. The data protection officer reviewed the proposed project regarding observance of the data protection law and gave permission to perform the study. All the data obtained were processed and evaluated anonymously and in compliance with EU's General Data Protection Regulation.

## Results

### Progress Testing in European Veterinary Medical Education

Of the 82 European Universities, we received 22 responses, equivalent to a response rate of 27 %. However, only 14 of these questionnaires could be evaluated (17%).

The survey revealed that in Europe, only Utrecht and the German-speaking countries used PTs. Seven Universities indicated that they currently did not have any PT but that they wanted to establish progress testing. Five Universities denied performing progress testing. The two Universities stating that they had carried out PTs in the past were part of the cooperation in the German-speaking countries.

As we could not identify any other published literature on PTs in veterinary medical education in Europe, we assume that to the best of our knowledge there are currently only two European PTs in veterinary medical education—in the German-speaking countries and at Utrecht University.

### Progress Testing at Utrecht University According to Favier et al. (9)

In 2011/2012, the Dutch Faculty of Veterinary Medicine at Utrecht University was the first European educational institute which had carried out progress testing twice as a pilot study. After adapting their curriculum in 2007, the main question was whether progress testing could be adapted to veterinary medical education. In this pilot study, all students who had started their undergraduate bachelor studies between 2006 and 2009 were tested during their following master's program. Additional points to focus on in this study were whether PTs were sensitive enough to measure the increase in knowledge over a 6-month period and to what extent the growth of knowledge could be measured.

The test structure followed the Maastricht PT in medical education. After a multi-stage review process, 150 single best answer-MC items were selected by blueprint. Questions from the undergraduate subjects and from the master's program were weighted with 90:60. The basis for the bachelor question pool was the bachelor's degree curriculum. The questions for the master's question pool were specially created for these two tests. Due to the reform of the curriculum, the students were separated into only two groups—before and after 2007. The participation for the years before 2007 was non-compulsory because those students could only participate in one PT. In contrast, participation for those studying after 2007 was compulsory.

The analysis of the PT was formative, not like the Maastricht role model, and the individual results were only sent to the students. All questions had an additional “don't know” option.

The score was calculated with one point for every right answer, with one point being deducted for every wrong response (formula scoring). The “don't know” option was not given a rating.

The results showed that PTs fitted the quality criteria and were able to present the increase in knowledge, though carrying out PTs at least three times a year would be more sensitive. The Utrecht PT showed good validity and students rated it as a good tool. They “perceived progress testing as relevant for their future practice.” The students were grateful for the feedback on their strengths and weaknesses as well as their performance level in general. For the students, the questions seemed difficult but the “don't know” option had a neutral effect on them. Favier et al. (9) pointed out that progress testing is suitable for a curriculum with a large amount of species as in veterinary medical education. Progress testing is a very variable tool and therefore completely independent of different curricula.

### Progress Testing in Veterinary Medicine in the German-Speaking Countries

The PTT was developed and implemented in 2013 by the Competence Centre for E-Learning, Didactics and Educational Research in Veterinary Medicine (KELDAT) supported by the assessment-group of the Charité-Berlin University of Medicine (Charité-Universitätsmedizin Berlin) (8). KELDAT was a project cooperation of all German-speaking institutes for veterinary education in Germany, Austria and Switzerland (D-A-CH) funded by the Volkswagen and Mercator Trusts (27).

The PTT was developed during the project duration from 2012 to 2016. To show the long-term development of increased knowledge, the PTT was carried out annually at five out of seven D-A-CH locations and at all seven locations in 2014. Further changes to the platform and the analysis were carried out to optimize the PTT for the period from 2017 to 2019, e.g., marker questions were established. Since then, the PTT has taken place at six locations, namely at every German University of veterinary medicine and additionally at University of Veterinary Medicine, Vienna, Austria (D-A).

The European Association of Establishments for Veterinary Education (EAEVE) defined a list of “day 1 competencies” for veterinarians as approved by the European Coordination Committee for Veterinary Training (ECCVT) (28). Evaluating, promoting and developing the quality and standard of veterinary medical faculties in Europe are the aims of the EAEVE (29). These “day 1 competencies” are reflected in the PTT content (8).

The PTT is a formative interdisciplinary test, meaning that the results are non-graded and have no influence on the further course of studies (8). The decision to make the PTT formative is based on the aim to implement a tool to examine the students' knowledge independent of the learning strategies (8). Thus, the PTT as a feedback tool enhances self-monitored learning and perhaps greater attention is given to teaching methods (27).

#### PTT—Test Construction

The PTT, which has the same set of questions for every student and is independent of the year of training, consists of 136 MC questions in five modules and 34 subjects—based on the given blocks from the EAEVE (Table 1) (8, 27). For each of the 34 subjects, four questions are chosen. In the style of Blooms Taxonomy as described in (30), two questions are on the taxonomy level “remember” and two on the taxonomy level “understand/apply.”

**Table 1:** Blueprint of the PTT, modified to Siegling-Vlitakis (8).

**Module**	**Subject**	**Subjects per module**	**Number of questions**
Basic subjects	Physics, chemistry, animal biology, plant biology, biomathematics	5	20
Basic sciences	Anatomy, histology/embryology, physiology, biochemistry, pharmacology/pharmacy/toxicology, virology, microbiology, immunology, epidemiology, parasitology	10	40
Clinical sciences	Pathology, obstetrics/reproduction and reproductive disorders, clinical medicine in horses/ruminants/small mammals, surgery incl. anesthesiology in horses/ruminants/small mammals, clinical medicine and surgery incl. anesthesiology in other species, radiology, veterinary legislation and forensic medicine, propaedeutic	12	48
Animal production	Genetics, animal nutrition, animal husbandry and agriculture, veterinary hygiene, animal ethology and protection	5	20
Food hygiene	Inspection and control of animal foodstuffs or foodstuffs of animal origin, food hygiene and technology	2	8

**Figure 1:**
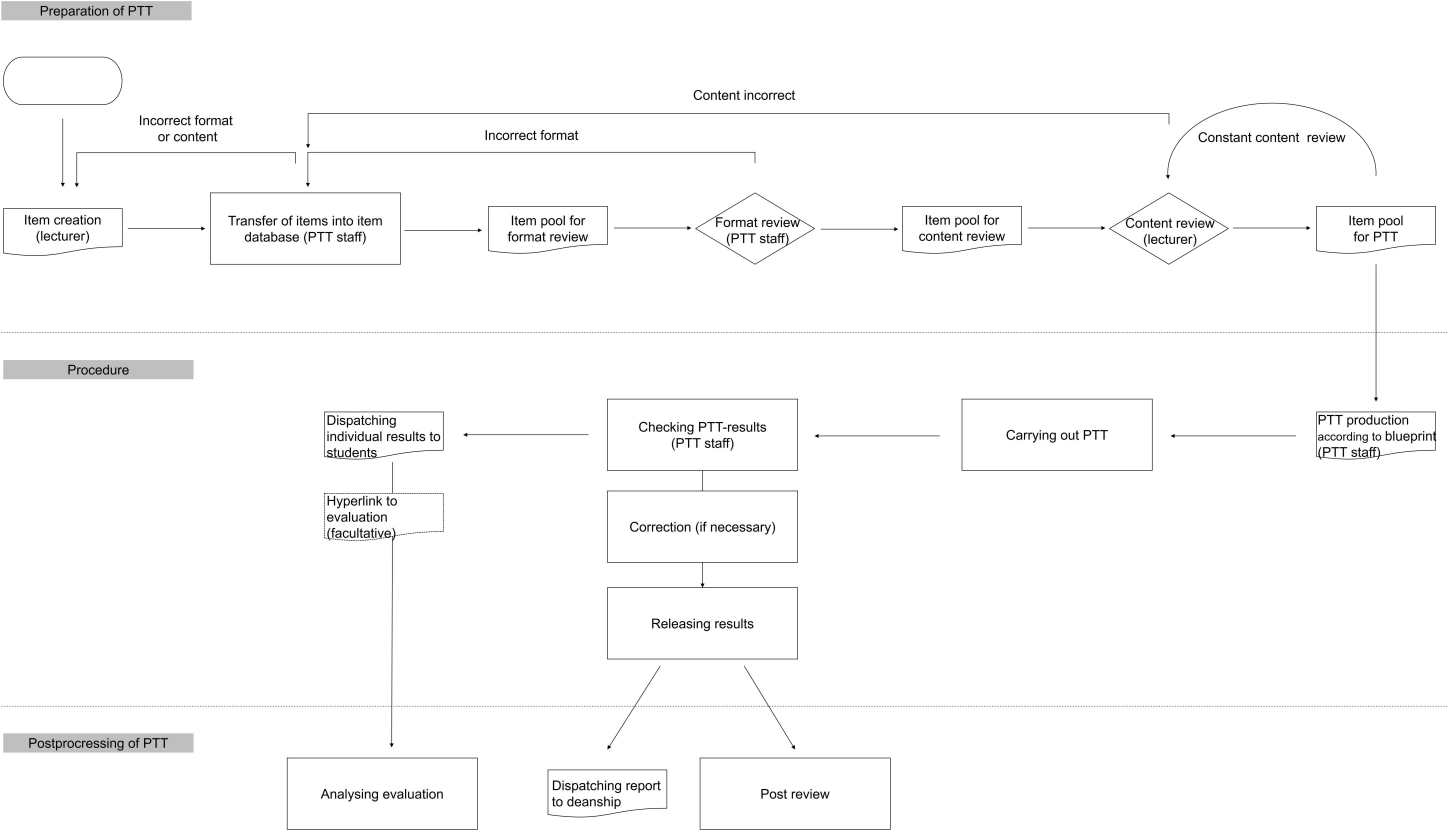
PTT workflow (Progress test veterinary medicine of the German-speaking countries).

The PTT set of questions changes every year. Every lecturer from the participating Universities has the possibility of writing question items (8). Afterwards, every item must be reviewed several times (8). The question administration and the procedure are done electronically. At every University, the local PTT members of staff enter the items using his or her identity in the management platform Q-Exam® Institution (IQul GmbH, Bergisch Gladbach, Germany) so the authors of the questions remain anonymous. Then a formal check is carried out by a local person responsible for the PTT. When an item fulfills the formal requirements, it is included in the content review. At least one accord with approval is necessary to move a question into the final PTT item pool. As there is a periodical review, every item can be moved to the format review or the content review again (Figure 1).

The electronic test is an A-Type MC test (single best answer) with four options. Additionally, there is always a “don't know” option to help create a realistic overview of knowledge. Therefore, this option is not rated at all. To calculate the individual results, formula scoring with one point for every right answer and one point deducted for every wrong answer is used.

#### PTT—Test Implementation

Until 2016, the participation was voluntary or compulsory for every student depending on the location, but the setting has changed. Since 2017, the PTT is voluntary at each partner University. Furthermore, online access was established through digital changes so that the test can now be taken at home. Every University is free to choose its setting and system (Table 2).

**Table 2:** Different settings for the PTT (since 2017).

**University**	**Platform for the test execution**	**Platform for test analysis and releasing of results**	**Location**	**Conditions of participation**	**Test execution**
1	Q-Examiner ^®^ IQuL	IQuL GmbH	Online externally	Voluntary	2017, 2018, 2019
2	Q-Examiner ^®^ IQuL	IQuL GmbH	Online externally	Voluntary	2017, 2018, 2019
3	Q-Examiner ^®^ IQuL	IQuL GmbH	Online externally	Voluntary	2017, 2018, 2019
4	UCAN	UCAN	Online externally	Voluntary	2018, 2019
5	ILIAS/UCAN	ILIAS, manual post-processing and releasing of results	Online externally	Voluntary	2017, 2019
6	LPLUS	In-house development	Online externally or internally in the E-Examination Centre	Voluntary	2017, 2018, 2019

*ILIAS, ILIAS open source e-Learning e.V. (Integriertes Lern-, Informations-, und Arbeitskooperations-System, Cologne, Germany); LPLUS (LPLUS GmbH, Bremen, Germany); Q-Examiner ^®^ IQuL, Institute for Quality Management in Teaching an Training (Bergisch Gladbach, Germany); UCAN, Umbrella Consortium for Assessment Networks (Institut für Kommunikations- und Prfungsforschung GmbH, Heidelberg, Germany)*.

The actual question administration and both review processes take place electronically in Q-Exam Institutions (IQuL GmbH, Bergisch Gladbach, Germany). This platform is used for creating the PTT, the review process and organization of the online database for the questions. After compiling the test by means of a blueprint, the PTT file is accessed from each location.

Taking the PTT is possible during a specified time span of 2 to 3 weeks in December, but every location chooses its own testing period. The maximum test duration is limited to 4 h. Immediately after finishing the test, the students receive their score with the total number of right, wrong and “don't know” answers.

#### Students' Feedback

Although coordinating and setting up the PTT is a collective project, comparison between the partner Universities is not appropriate due to the different curricula. This means that the deanery and every student only receive information comparing the performance and feedback on their own University's PTT. Each location evaluates the results of its own students and does not compare them with those of the partner Universities. For this reason, no statistical parameters can be specified.

**Figure 2:**
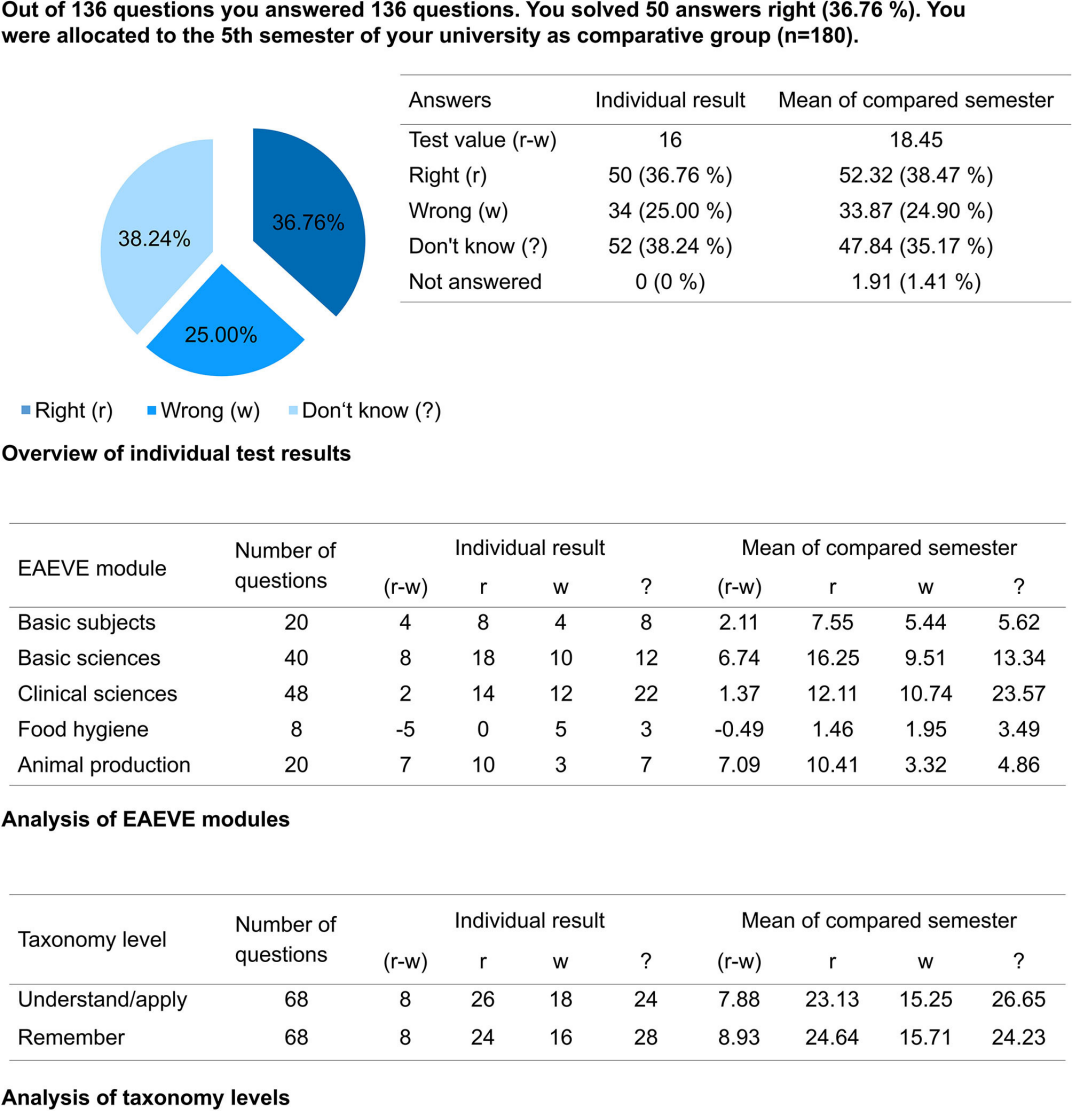
Individual total test result, exemplary presentation of a fifth-semester student; EAEVE, European Association of Establishments for Veterinary Education; r, right answer; w, wrong answer; ?, “don't know” option.

**Figure 3:**
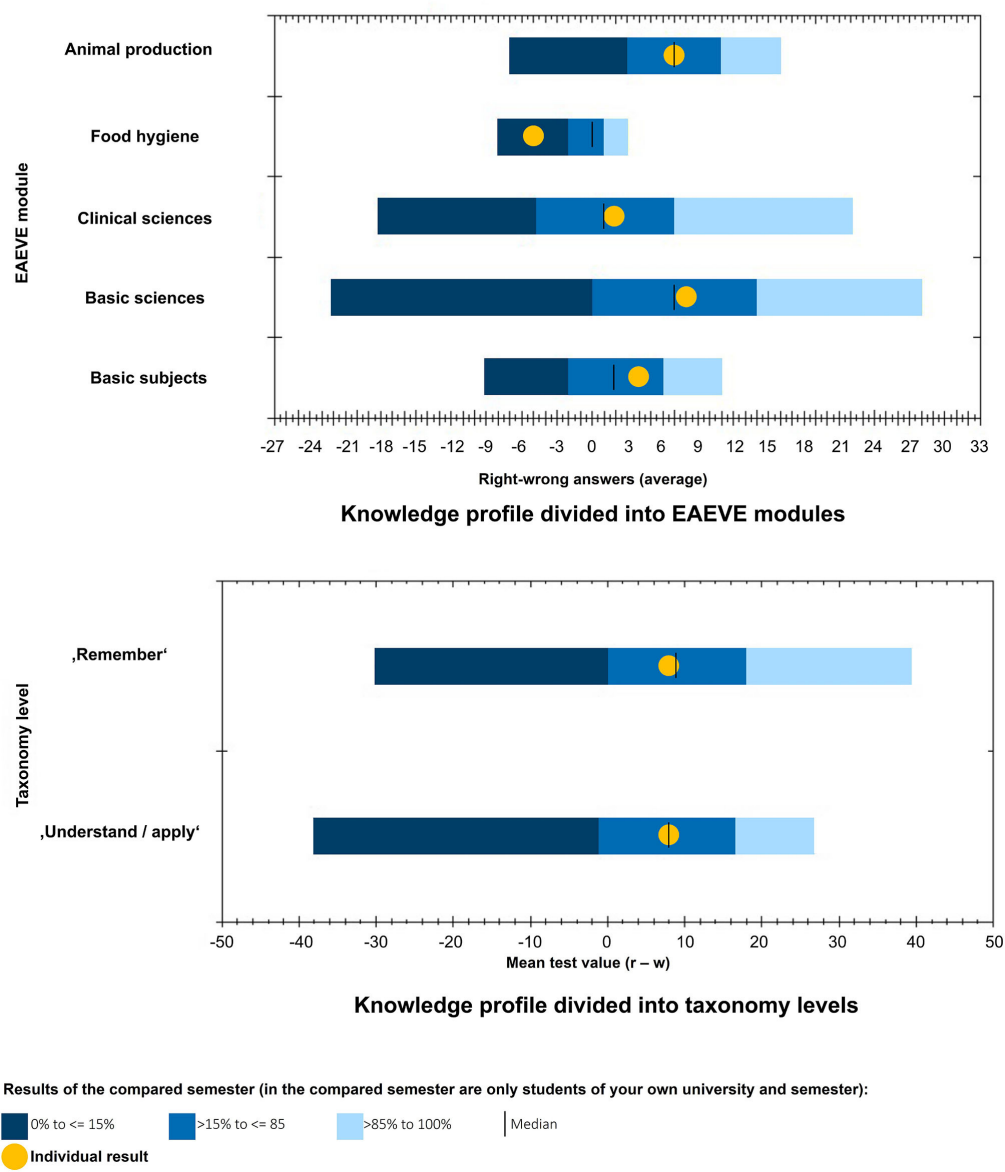
Knowledge profile divided into EAEVE (European Association of Establishments for Veterinary Education) modules and into taxonomy levels; exemplary presentation of a fifth-semester student; r, right answer; w, wrong answer.

**Figure 4:**
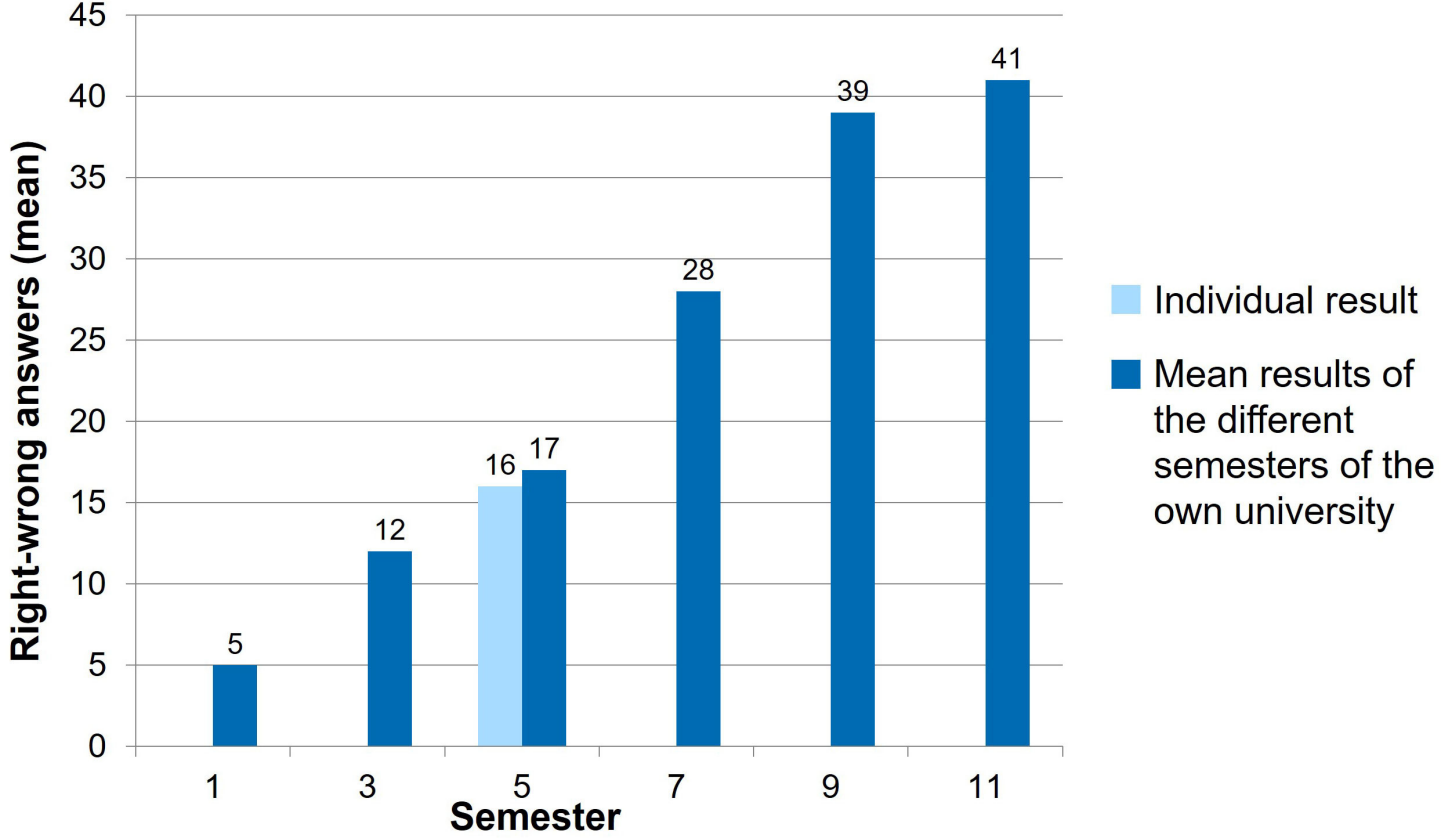
Level of knowledge of each semester, exemplary presentation of a fifth-semester student.

**Figure 5:**
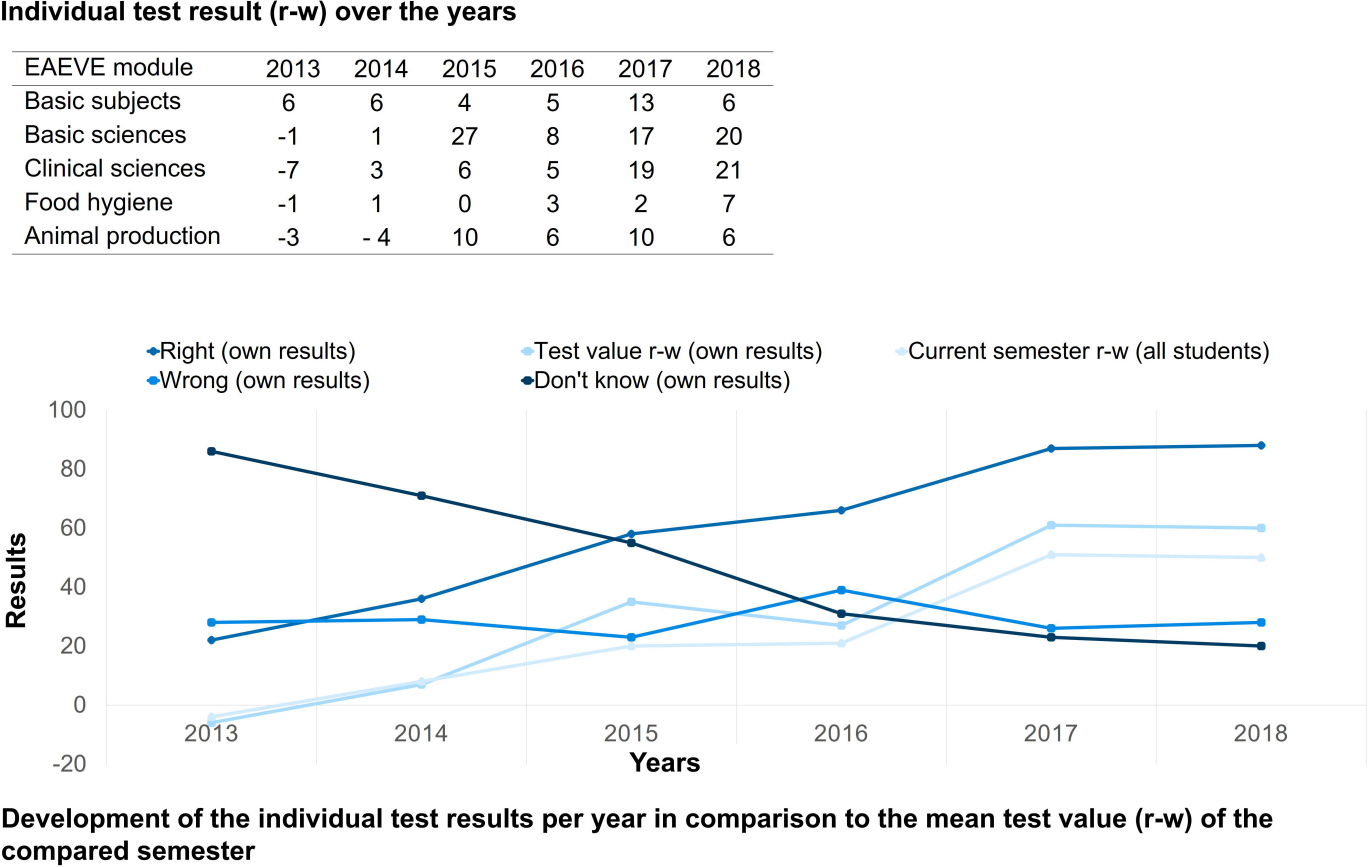
Individual test result over the years (for the students also available as graphics) and progress display of individual results and compared semester, exemplary presentation of an eleventh-semester student; r, right answer; w, wrong answer.

The students first receive immediate brief preliminary feedback in the form of their final score directly after finishing the PTT. After some time, they receive detailed individual feedback. The results are then analyzed in accordance with the utilized system. All students receive their individual score, a breakdown of EAEVE modules and the taxonomy level (Figure 2), in addition to a complete result overview of each subject. Their individual performance in a semester is additionally presented and how the students performed in the PTT in relation to fellow students in that semester (Figure 3). Finally, the average test score of each semester is compared (Figure 4). As from the second participation in the PTT the students receive a conclusion of their individual test results and in comparison to their semester over the years (Figure 5). By means of this analysis, the students realize their strengths and weaknesses and are compared to their fellow students (8). Since 2017, an overview is sent automatically to the deanery if Q-Examiner® is used. This includes the number of participants in the last PTT but also over the years and an overview of right (r) and wrong (w) answers and the “don't know” (?) option.

### PTT Evaluation

The link to the evaluation of the last PTT in December 2018 was sent to the students from four of the six partner Universities. The evaluation was available from 15 May to 14 October 2019.

**Figure 6:**
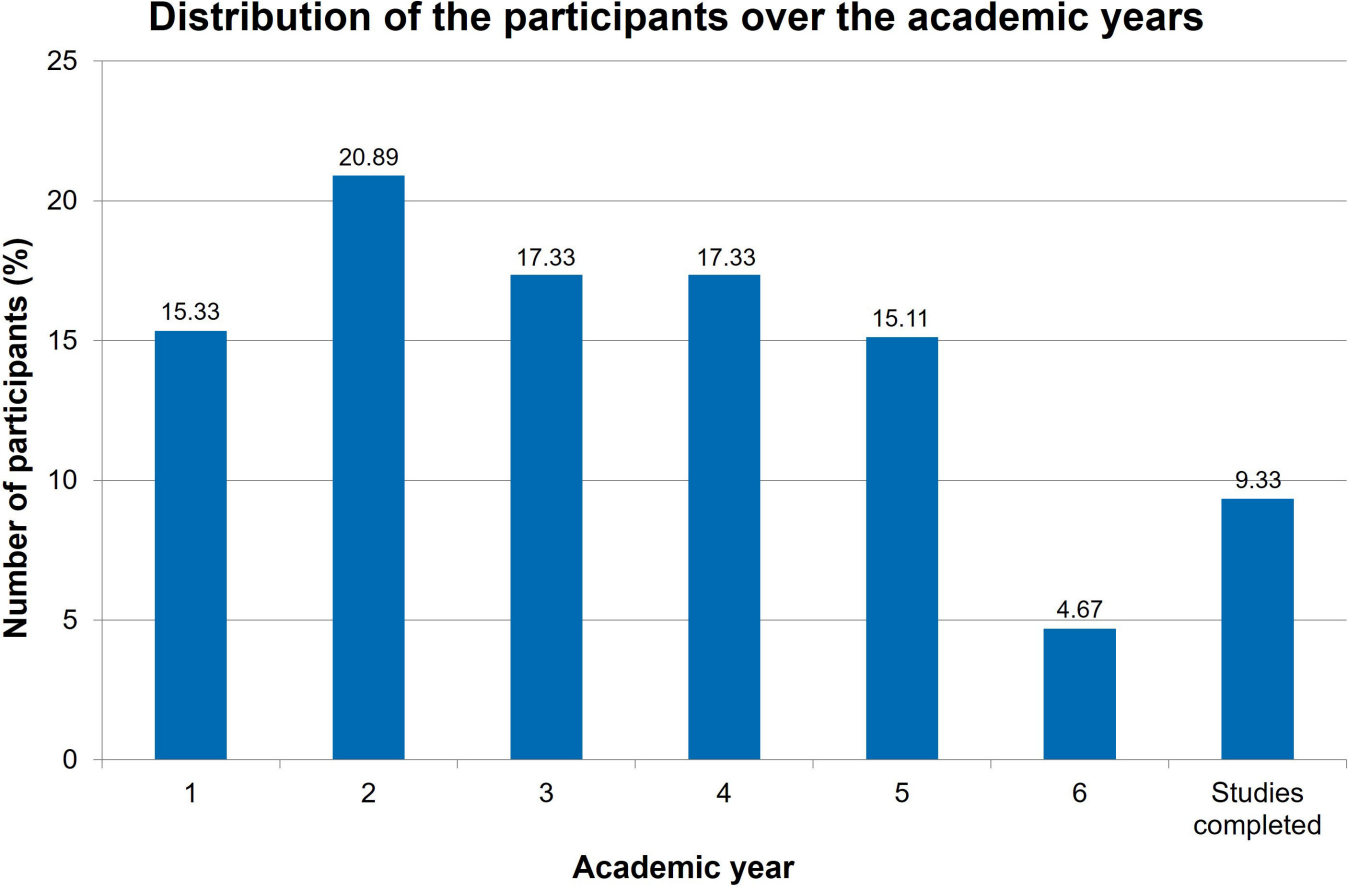
Evaluation: Distribution of the participants in the survey over the academic years (*n* = 450).

During this period, of the 567 students from four Universities who opened the online survey, 450 of them completed the questionnaires. The distribution of the participants over the academic years is shown in Figure 6.

In December 2018, 336 of the respondents (74.67%) took part in the PTT. The non-participants were allowed to give reasons for not carrying out the test: 21 of the respondents (4.67%) indicated a lack of time, lack of interest or too much effort involved in carry out the PTT and 19 students (4.22%) simply forgot to do the test. Eleven students (2.44%) did not participate in the PTT in 2018 because they were not informed about the PTT at that time or the information given was unsatisfactory. In addition to those who did not express any interest, seven students (1.56%) were busy with revising for their exams. Four respondents (0.89%) specified technical problems with their computer.

To the question if the students had carried out previous PTTs, 11 respondents (2.44%) affirmed carrying out the first test in December 2013. Forty-one students (9.11%) participated in 2014, 79 (17.56%) in 2015, 138 (30.67%) in 2016 and 208 (46.22%) in 2017.

**Figure 7:**
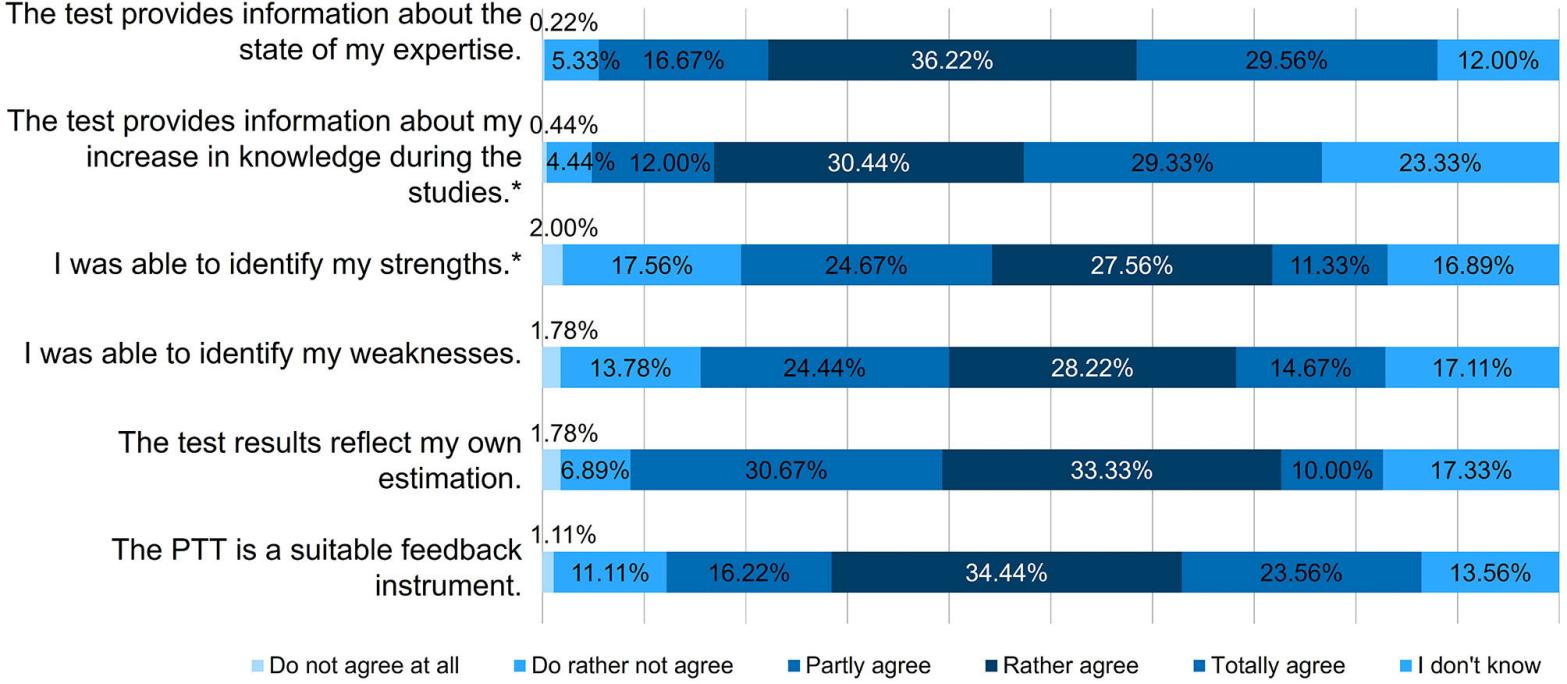
Evaluation: Rating of the PTT (*n* = 450); PTT, Progress test veterinary medicine of the German-speaking countries. * = Due to correct rounding, the total is not exactly 100%.

For the evaluation, the PTT should be assessed with a Likert scale (Figure 7). The Chi^2^-test was chosen for the non-parametric analysis to test the null hypothesis “There is no association between the year of study and the form of responses.” For the following statements (Figure 7) this null hypothesis was accepted (*p* > 0.05): “The test provides information about the state of my expertise.” (*p* = 0.5375), “I was able to identify my weaknesses.” (*p* = 0.0939), “The PTT is a suitable feedback instrument.” (*p* = 0.1088).

For the remaining three statements in Figure 7, *p*-value indicated a significant association (*p* < 0.05) and the null hypothesis was rejected.

For the statement “The test provides information about my increase in knowledge during the studies” *p*-value was 0.0006. It was noticeable that significantly more students from the first year of study answered with “I don't know” (Cell Chi^2^ = 12.001), but this answer option was chosen significantly less by the fifth year students (Cell Chi^2^ = 3.9003). In year 4, a particularly large number of students answered with “Do not agree” (Cell Chi^2^ = 4.5966) and “Partly agree” (Cell Chi^2^ = 3.3985).

For the statement “I was able to identify my strengths” *p*-value was 0.0077. This time the second year of study chose the option “I don't know” significantly more often (Cell Chi^2^ = 4.1578). On the other hand, a particularly large number of students in the third year answered with “Partly agree” (Cell Chi^2^ = 7.188) and the fourth year of study was again found to select “Do not agree” significantly more often (Cell Chi^2^ = 2.9841). In contrast to the above statement, the option “Partly agree” was chosen significantly less often in the fourth year of study (Cell Chi^2^ = 3.529).

For the statement “The test results reflect my own estimation” *p*-value was 0.0476.

Here significantly more first year students agreed with this statement (Cell Chi^2^ = 2.1943) and significantly less chose the option “Partly agree” (Cell Chi^2^ = 3.9653).

It was noticeable in the second year of study that significantly more students chose “I don't know” (Cell Chi^2^ = 3.6452), but also here significantly fewer students voted “Partly agree” (Cell Chi^2^ = 2.125).

The fourth year of study—as in the first statement—again chose significantly more often “Do not agree” (Cell Chi^2^ = 2.6594) and “Partly agree” (Cell Chi^2^ = 2.0956) and the 5th year of study also voted conspicuously more often for the option “Partly agree” (Cell Chi^2^ = 2.4492).

**Figure 8:**
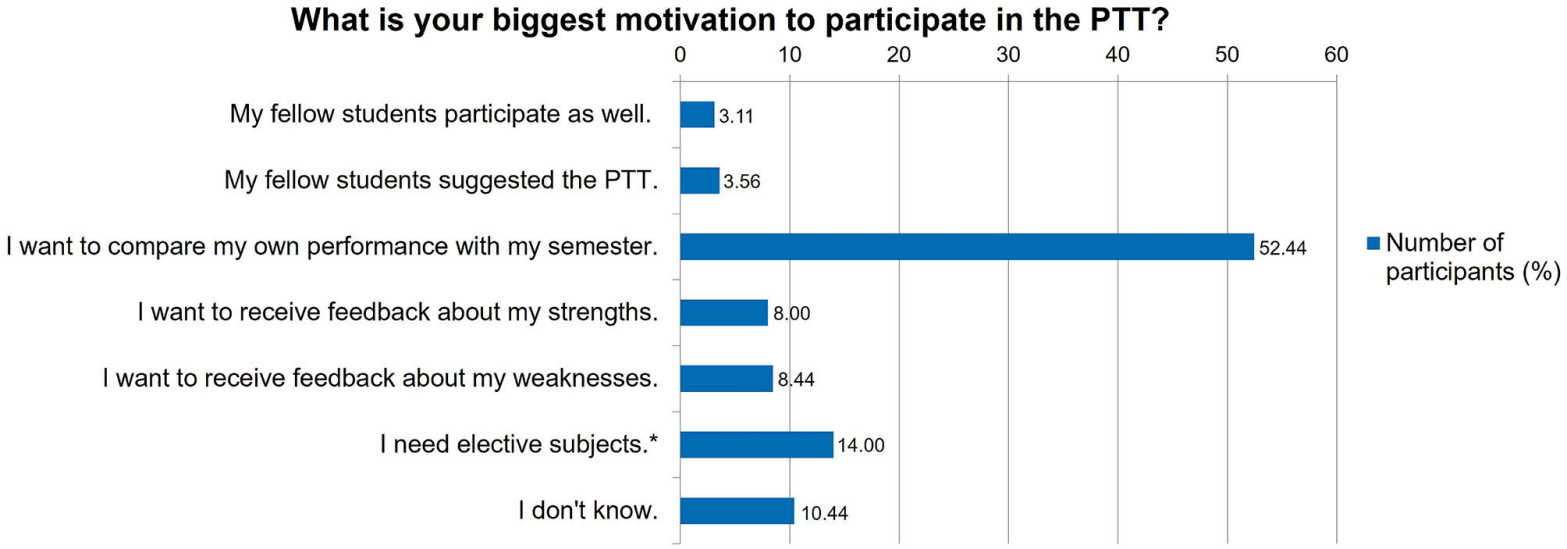
Evaluation: Reasons for participating in the PTT (Progress test veterinary medicine of the German-speaking countries) (*n* = 450). * = Only students from the TiHo had the option “I need elective subjects”.

Later in the evaluation the reasons for participating in the PTT were identified (Figure 8). For this question the null hypothesis was also accepted (*p* = 0.0835).

Student satisfaction regarding feedback opportunities during studies is shown in Table 3A. Thereby, an option for comments was given (Table 3B). Here, no association between the year of study and the answers to the feedback opportunities could be detected (*p* = 0.5944).

**Table 3:** **(A)** Evaluation: Satisfaction with the given feedback opportunities in the studies (*n* = 450) and **(B)** comments (*n* = 45).

	**Number of participants**	
	***n***	**%**
**(A) Are you satisfied with the given feedback opportunities during the studies regarding your own performance? (*****n*** **=** **450)**		
Yes	192	42.67%
Partly satisfied	160	35.56%
No	38	8.44%
I don't know	60	13.33%
**(B) Comments (*****n*** **=** **45)**		
**Comments about the PTT**		
- Appreciation of the PTT	8	17.78%
- PTT is not/hardly meaningful	10	22.22%
- Preclinical years demotivated, PTT not meaningful yet	7	15.56%
- No sustainable knowledge	5	11.11%
**Comments about the studies**		
- Request for more feedback from the lecturers	8	17.78%
- Teach more interdisciplinary relations	3	6.67%
- More professional relevancy	2	4.44%
- Inadequate grading system	2	4.44%

Finally, the students were asked if they planned to participate in the next PTT in December 2019. This was affirmed by 347 respondents (77.11%); only 16 students (3.56%) refused to participate further. For fifty participants (11.11%), they will have completed their studies by then. Thirty-seven respondents (8.22%) selected the “I don't know” option.

## Discussion

Though PTs offer many possibilities [e.g., showing spontaneously accessible knowledge (1), measuring the increase in knowledge (14), serving as management (1) or comparative tool for curricula (3)], our study has been shown that progress testing is uncommon in European veterinary medical education. However, PTs involve time and effort so a cooperation in implementing and performance as in German-speaking countries can be a solution.

The PTT was carried out as part of a research project at the outset. This project finished in 2016, but progress testing was so convincing that it was still used in German-speaking countries afterwards. The initial aim of progress testing was to implement a suitable feedback instrument for students to support self-directed learning and to improve the quality of training in veterinary medicine.

The results of the survey show that the participation of surveyed students in the PTT had risen continuously since its implementation in 2013. This underlines that students have accepted this feedback tool. The main reason for the students to participate in the test was to compare their own performance with fellow students in their semester (Figure 8), which is in contrast to another study showing that students use PTs to measure their increase in knowledge (14). Especially students of the fourth year of current study rejected the statement that the PTT was useful to detect their increase in knowledge (Cell Chi^2^ “Do not agree” = 4.5966 and “Partly agree” = 3.3985). The first year of study was strikingly unsure about this statement (Cell Chi^2^ “I don't know” = 12.001). However, the students particularly agree that the PTT provides information about the state of their expertise and the increase in knowledge during the study (Figure 7).

Overall, it is particularly noticeable that the fourth year of study generally takes a more negative attitude toward the three statements with *p* < 0.05 than the remaining years of study. Further studies would be needed to check whether this effect also occurs in other cohorts and to explore possible explanations.

Unfortunately, 14.00% replied that they only participated in the PTT for taking mandatory points in elective subjects (Figure 8). As only a single University had this opportunity, it is remarkable that the need for elective subjects is the second leading option for carrying out the PTT.

The distribution of the participants in the survey showed a remarkable small amount of respondents in the sixth year of study (Figure 6). This can possibly be explained with the fact that the final exams occur nearly simultaneously in the sixth year of study.

Despite—or perhaps precisely because of—the digital age some technical problems impeded a participation in the PTT, since students missed technical support at home. In contrast during electronic assessments in a lecture hall at University such support is constantly available.

Figure 7 shows that more than 65% of the respondents rather and totally agreed that the PTT provided information about the state of expertise. Nearly 60% rather and totally agreed that PTT provided information about their increase in knowledge during the studies and that the PTT was a suitable feedback instrument (Figure 7). Even though 15–20% of the respondents replied that the PTT did not identify neither their strengths nor their weaknesses the majority proved a high acceptance of the PTT (Figure 7). In addition, about three quarters of the respondents were partly satisfied and satisfied with the given feedback opportunities in the studies (Table 3A).

Overall, the PTT as a feedback tool received a large acceptance over the years. Of course, also negative views on the PTT exist (see Table 3B). One reason for rejecting the PTT could be the assumption that a theoretical test was not suitable to examine practical know-how. Furthermore, the PTT could demotivate students from preclinical years because their level of knowledge is small (4). Nevertheless, more than three quarters of the respondents planned to participate in a forthcoming PTT.

As described in Table 2, all German-speaking Universities participating in the PTT were using different settings for the test execution as well for the analysis and releasing of the results. This is disadvantageous because the analysis and results have a poor comparability. Since the Universities did not want any comparison among each other due to different curricula, this problem was negligible. In addition, the variety in the settings demonstrated that progress testing could be introduced and performed individually and is independent from any platforms.

Whether the PTT is a suitable measuring instrument should still be examined more closely. Therefore, an additional study is planned for a detailed analysis of the PTT. According to (8), this study will clarify how the PTT can also be used for quality management based on the following questions:

1. Is an increase in knowledge over the years detectable?
2. Is the growth in knowledge dependent on the curriculum?
3. Is there long-term knowledge internalization?
4. Are the taxonomy levels “remembering” and “understand/apply” at the same level?
5. Which level of knowledge do the students achieve in particular subjects?
6. Are marker questions suitable for checking the test stability?

Implementing progress testing involves a great deal of effort. For this reason, a checklist for implementing progress testing is included (Table 4).

**Table 4:** Checklist with key questions and tasks for implementing progress testing after identifying the main goals (e.g., having a feedback instrument or comparing old and new curricula).

1. Do you want to organize your PT in collaboration with partner Universities or as a single University project? 2. Where do you want to collect the items? Do you need an examination management platform? 3. Do you want to perform your PT as a paper-based test or as an electronic test? 4. Decide which software you need to perform your PT and to evaluate the results. 5. Which content do you want to cover with your PT? Which content should be the basis for your test blueprint to achieve a high test validity (31)? 6. Which taxonomy level should the questions reach? 7. What should be the total number of questions and how many questions do you want per subject to achieve good reliability? 8. Which question type do you want to use for your PT–single best answer multiple-choice questions, true/false questions or any other question type? 9. Create the selection criteria for the questions. 10. How do you want to perform your formal review and review of content? 11. Which students or semesters should form the target group? 12. Should participating in the PT be compulsory for students or on a voluntary basis? Should this depend on the University year? 13. How often and at which time of year should the test take place? 14. How should the results and analysis have been recorded? a. Who should have access to the results and analysis? b. Decide on the design of the analysis, including graphics c. Which export file is necessary? d. Determine working steps for the post review. 15. Who should have access to the results? Which data should be published?

## Data Availability Statement

All datasets generated for this study are included in the article/supplementary material.

## Ethics Statement

This study was conducted according to the ethical standards of the University of Veterinary Medicine Hannover, Foundation. The doctoral thesis committee of the University, which acts as the University's ethics committee, validated the project in accordance to ethical guidelines regarding research with human participants and approved the study. Written consent to be part of the study was obtained from all participants.

## Author Contributions

LH and ES conceived and designed the study. ES, AT, and JE supervised the study. LH, ES, and AT created the questionnaire for the EU survey. ES, BP-M, DB, SB, CB-R, and IP created the questionnaire for the evaluation. LH collected, analyzed, and interpreted the data, which was supervised by ES. The manuscript was drafted by LH with critical input from all the other authors and notable revisions from ES and AT. All authors reviewed and approved the final version.

## Conflict of Interest

The authors declare that the research was conducted in the absence of any commercial or financial relationships that could be construed as a potential conflict of interest.

## Abbreviations

EAEVE: European Association of Establishments for Veterinary Education
MC: multiple choice
PT: progress test
PTM: German medical progress test
PTT: Progress test veterinary medicine of the German-speaking countries.
